# Unravelling the threads: understanding the interplay of Cultural values, female workforce engagement, human development index and suicide rates

**DOI:** 10.1007/s00737-024-01502-9

**Published:** 2024-08-29

**Authors:** Elisa Vigna, Ingrid van Balkom, Michaeline Bresnahan, Keely Cheslack-Postava, George Musa, Megan Ryan, Norbert Skokauskas, Christina Hoven, Vladimir Carli

**Affiliations:** 1 National Centre for Suicide Research and Prevention of Mental Ill-Health (NASP), Karolinska Institutet, Stockholm, Sweden; 2 Jonx, department of (Youth) Mental Health and Autism, Lentis Psychiatric Institute, Groningen, Netherlands; 3 Rob Giel Research Centre, Department of Psychiatry, University Medical Center Groningen, Groningen, Netherlands; 4 Department of Epidemiology, Mailman School of Public Health, Columbia University, New York, US; 5 Global Psychiatric Epidemiology Group, Division of Child and Adolescent Psychiatry, Department of Psychiatry, New York State Psychiatric Institute, New York, US; 6 Department of Mental Health, Regional Centre for Child and Youth Mental Health and Child Welfare (RKBU Central Norway), Faculty of Medicine and Health Sciences, Norwegian University of Science and Technology, Trondheim, Norway

**Keywords:** Suicide, HDI, Female labor participation, Individualism, Collectivism

## Abstract

**Purpose:**

Suicide is a major public health problem across the world. Extensive research on the field shows that suicide is affected by several sociological, economic, and cultural risk factors. Over the last century, social changes have driven the reshaping of traditional gender roles, often in an uneven fashion, strongly depending on context. This study proposes updated findings on the impact that changes in traditional gender roles could have on suicide rates

**Methods:**

It will do so by examining the correlation between female labor force participation (FLPR) and sex-specific suicide rates. Moreover, it will examine this association depending on human development (HDI) and Hofstede’s individualism index. To do so, data from 2010 to 2019 from 47 countries is collected from the WHO, ILOSTAT and UN agencies’ websites.

**Results:**

Analysis show a significant interaction between FLPR, HDI and individualism index scores on male suicide rates (*p* = 0.002). There is a negative association between FLPR and male suicide rates in relatively lower HDI countries, while in very high HDI countries an increase in FLPR is correlated with an increase in male suicide rates. Similar trends but no significant interaction is observed for female suicide rates.

**Conclusion:**

This study suggests that female participation is beneficial for male population as it reduces male suicide rates. However, this association appears to be context dependent. In countries where institutional adjustment is already established, and human development is very high, other factors might be of interest in examining the trends of suicide rates among men and women.

## Highlights

- This article examines the correlation between female labor force participation (FLPR) and sex-specific suicide rates, depending on human development (HDI) and Hofstede’s individualism index, in 47 countries between 2010 and 2019.
- In countries with mean HDI and lower HDI, high female participation in the workforce was associated with a declining trend in male suicide mortality.
- Individualism/collectivism scores alone had no significant effect on male suicide trends.
- No significant results were recorded for female suicide rate.
- Variations due to country context remain highly significant, limiting the generalization of results.

## Introduction

The relationship between economic participation and mental health outcomes has remained a core concern among public health researchers (Burr et al. 1997; Chen et al. 2012; Milner et al. 2012). Central to this exploration is the phenomenon of suicide, as the reflection of psychological, social, and economic pressures, among other factors (Wray et al. 2011). Globally, in 2019, 1.3% of deaths were from suicide, with higher rates for males (12.6 per 100.000) than females (5.4 per 100.000) (WHO 2021). Among the factors affecting sex differential in suicide rates, female labor force participation stands central. Over the last century, female economic participation has significantly changed, increasing in many regions of the world, reflecting changes in gender norms, economic necessities, and women’s empowerment (Thévenon 2013). This article delves into the complex interplay between changes in female labor force participation and men’s and women’s suicide rates.

On a theoretical level, the study of gendered suicide trends is traced to Durkheim’s 1897 work. Durkheim suggested that women’s integration through domestic roles could be a protective factor against suicide. While he does not explicitly analyze the connection between women’s labor participation and suicide, later studies explored this idea, proposing that increased societal integration of women through work and politics should affect suicide rates. In 1964, Gibbs and Martin argued that women who enter the workforce become more likely suicide victims as the pressure of dual roles may lead to role overload, poor relationship quality, and, without support, deteriorating mental health (Ciciolla and Luthar 2019; Harryson et al. 2012). In contrast, Sieber (1974) and Marks (1977) proposed that employment could reduce stress by providing income and social support, supported by studies showing a link between higher income, better life conditions, and lower suicide rates (Howell and Howell 2008; Wray et al. 2011).

Recent theories have expanded to include contextual factors. Economic development influences female labor participation and its association with suicide, with industrialization initially reducing female labor (Boserup 1970; Goldin 1994; Tam 2011). In this context, social change driven by women wanting to enter the labor market disrupts traditional values, leading to uncertainty and role ambiguity, which in turn might produce an increase in suicides. However, as institutions adapt to social changes and the economy further develops towards expanding the service sector and improving work prospects, the rise in workforce participation could lead to beneficial effects of role enhancement (Pampel 1998).

The way development influences population health may be further moderated by cultural factors (Eckersley 2006; Mansyur et al. 2009). In this study, we examine the axis of individualistic/collectivistic cultural values proposed by Hofstede (2001). Previous studies show mixed findings on the topic. For instance, in cultures identified as collectivistic, individuals often experience mutual support, leading to reduced stress from challenging life events (Triandis 2000), along with a positive correlation between individualism index and suicide rates (Eckersley and Dear 2002; Rudmin at al., 2003). However, Lenzi et al., (2011) challenge the straightforward link between suicide rates and Hofstede’s individualism index. Similarly, other studies failed to find significant associations between suicide rates and measures of individualism (Schwarzenthal and Milfont 2016; Lester 2003).

Given the rapid evolution of recent economies and social changes, there is a need to update findings and shed more light on the role of culture in the relationship between female labor force participation and suicide rates. While existing literature has addressed this relationship, the results remain disputed, particularly regarding the impact of cultural values like individualism and collectivism. The aim of this study is, therefore, to explore the extent of the relationship between female labor force participation and suicide rates, including the possible impact of individualism index scores, as well as the level of human development. The broad hypothesis would be that female integration into the modern labor market may have diverse effects on suicide rates, depending on the contextual factors (i.e., human development), and that more collective societies will experience lower disadvantages from the role conflict and role strain introduced by female labor-force participation.

## Method

Data was collected for the period between 2010 and 2019, the latest available year for mortality data. Since not all countries have reliable data, the present study focused on countries where suicide data and data on the other variables under examination were available and reliable (in the “Sample” section).

### Outcome variable

Estimates of age-adjusted suicide rates were collected from the World Health Organization (WHO) mortality database (WHO 2023). Suicide rates per 100.000 habitants were collected for each year, country, and sex (male, female, both) separately. Vital registration data from each country used to compile the estimates refers to the ICD-10 codes X60–X84 and Y870.

### Explanatory variables

Female labor force participation (FLPR) was collected from the International Labor Organization Department of Statistics (ILOSTAT) database (ILOSTAT 2022). It is reported as the percentage of the female population aged 15 or more participating in the labor force each year (from 2010 to 2019) and in each country under study. The labor force participation rate is a measure of the proportion of a country’s working-age population that engages actively in the labor market. The measurement requires data of both employment and unemployment.

Human Development Index (HDI) was collected from the United Nations (UN) database for each year and country separately (United Nations Development Programme 2023). The HDI is the geometric mean of normalized indices for three dimensions: life expectancy at birth, education (including expected years of schooling and mean years of schooling), and income (Gross National Product (GNI) per capita in purchasing power parity in US$). HDI values range from 0 to 1, where 1 indicates the highest human development (Santos and Alkire 2011).

Individualism/collectivism index scores for each country were as calculated in Hofstede’s (2001) empirical analysis of cross-national data collected in the late 1960s on IBM employees, resulting in four basic cultural dimensions. As described by the author, the concept of individualism indicates a preference for a society characterized by a loosely connected social structure, where individuals are expected to primarily fend for themselves and their immediate families. In contrast, collectivism indicates a preference for a tightly interconnected social structure, where individuals anticipate support from their relatives or other group members (Hofstede 2001). Higher values indicate higher individualistic values, and lower values more collectivistic values. According to the data available in the Hofstede Insights Country Comparison Tool (Country Comparison Tool 2023), one score is attributed to each country for the entire time period under study.

### Sample

**Fig. 1:**
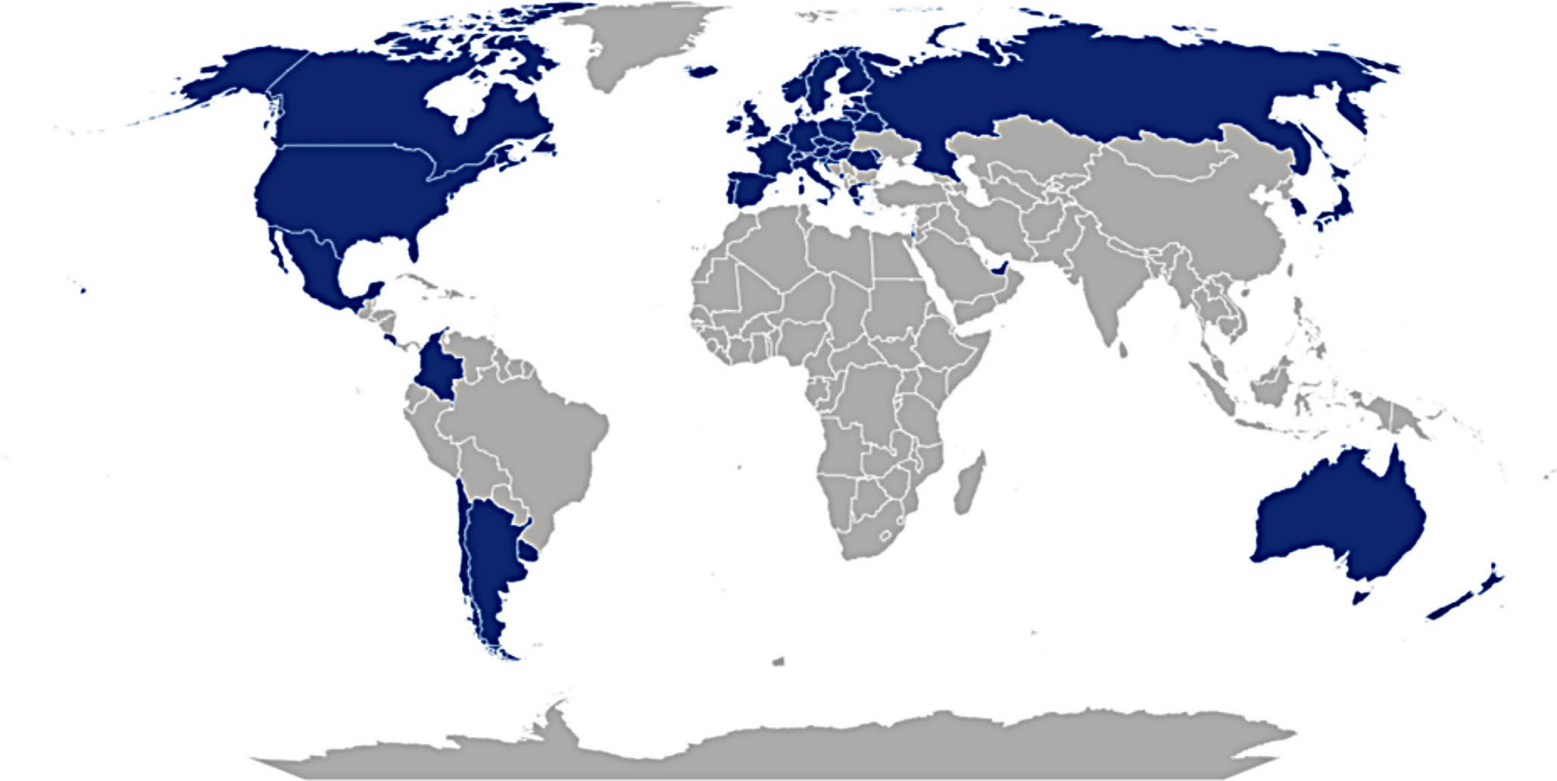
Map of the included countries (in blue)

The main inclusion criteria were high-quality death registration data (based on the WHO inclusion criteria for countries with high-quality death registration data) (WHO 2020). From the list of countries with high-quality death registration data, those with missing data on HDI, FLPR and individualism score and outliers (using interquartile range (IQR) criterion) were excluded. This resulted in a final sample size of 47 countries (Supplementary materials) (Fig. 1) distributed over four WHO world regions (Table 1).

**Table 1:** Sample distribution by WHO region

Region	Number of included countries
Americas	8
Europe	33
Western Pacific	5
Eastern Mediterranean	1
**Total**	**47**

### Statistical analysis

Linear mixed model analysis was conducted with the R package lme4 and lmerTest (the latter to extract p-values) in R statistical software (R version 4.1.0).

Separate analyses were conducted for male and female suicide rates. Log-transformed suicide rates were used in all three models to satisfy the normality assumption. In addition, explanatory variables were scaled and centered (Table 2).

**Table 2:** Mean and standard deviation of HDI and FLFP

	Mean	SD
FLFP	53.82	7.32
HDI	0.87	0.06
Individualism/collectivism score	52.83	22.27

For practical reasons, when discussing the results, values one standard deviation below the mean of HDI are defined as “lower HDI,” values around the mean as “mean HDI,” and values one standard deviation above the mean as “higher HDI.” The same was done for FLPR values.

Prior to the analysis, the random terms (country and time) were examined in relation to the outcome to assess whether they had random slopes and intercepts and whether these were correlated. Suicide rates were displayed by year for each country, revealing a heterogeneous sample in terms of slopes and intercepts. The coefficients were collected, and a correlation test was performed, demonstrating correlated intercepts and slopes (*p* < 0.001). Therefore, two linear mixed models with random correlated slopes (time-variable) and intercepts (countries’ names) have been applied.

The first model explored the interaction effects of HDI and FLPR on suicide rates. In the second model, the individualism/collectivism score was inserted (mixed model with a three-way interaction: FLPR * HDI * individualism/collectivism scores). Finally, the models were compared, and the goodness of fit was tested (Supplementary materials).

The presence of multicollinearity was evaluated by calculating the variance inflation factor (VIF), which gauges the influence of collinearity between variables in a regression model. None of the VIF scores surpassed the critical value of 3, indicating that multicollinearity did not pose a concern in our dataset (O’Brien 2007).

## Results

### Model 1

**Fig. 2:**
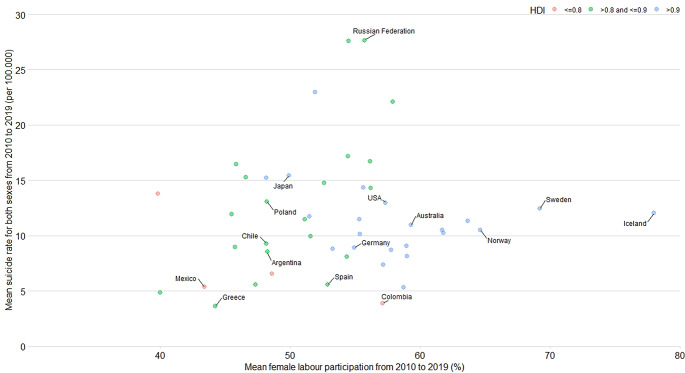
Mean female participation and mean suicide rates for both sexes, between 2010 and 2019 by country and HDI

Mean FLPR and suicide rates (both sexes) were extracted for each country for a preliminary visual inspection of the data. As displayed in Fig. 2, most of the countries in the sample had a mean FLPR that ranged from 40 to 60% participation. However, within this range, mean suicide rates varied. A positive association between FLPR and overall suicide rates was visible in countries with an HDI > 0.8 (and lower than 0.9), and a trend toward a negative association between FLPR and overall suicide rates was visible in countries with an HDI > 0.9 (Fig. 2).

**Fig. 3:**
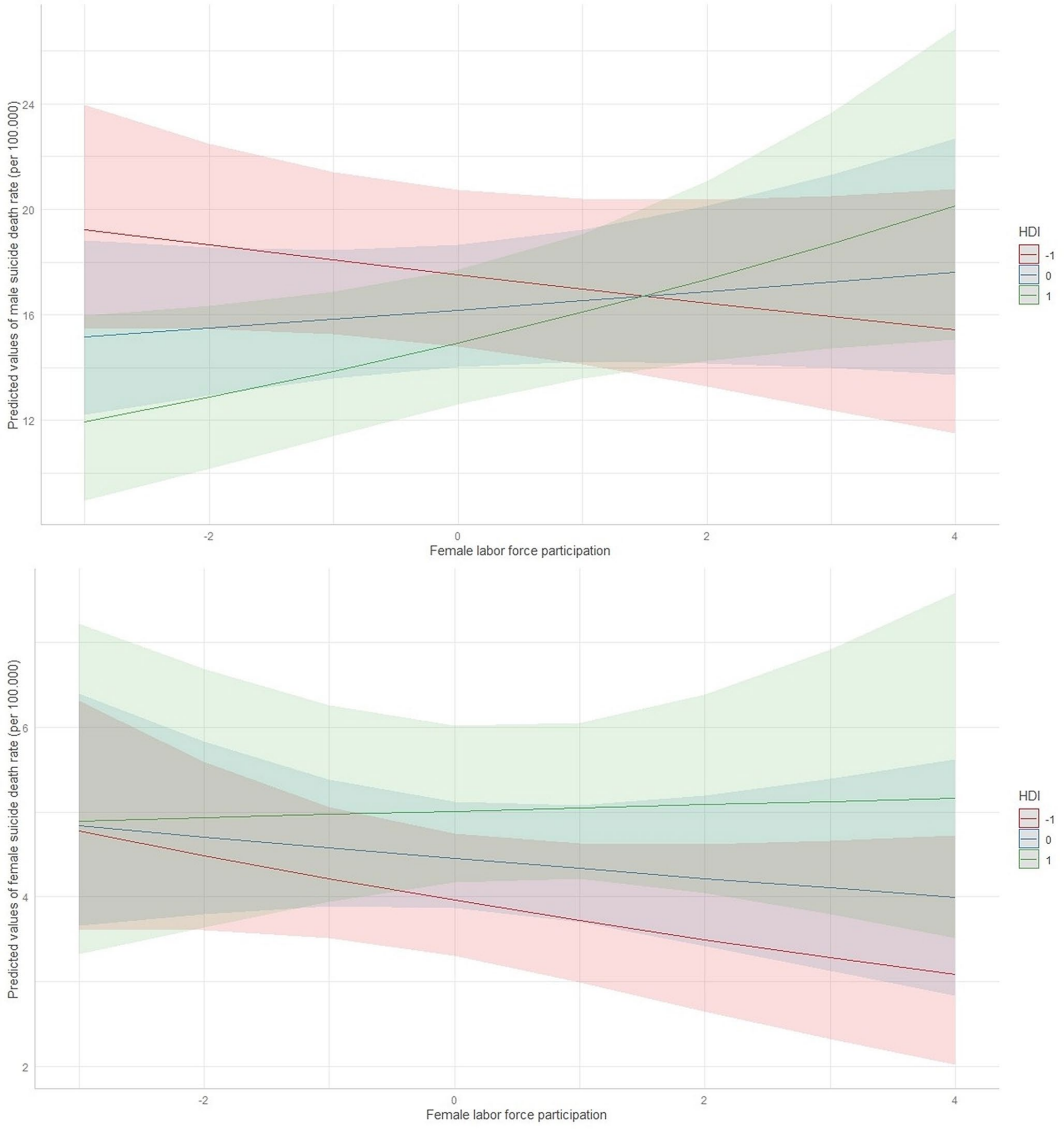
Predicted male (top) and female (bottom) suicide rate, by changes in FLPR (scaled) and HDI (scaled)

Results from the mixed model showed no significant main effect of FLPR (t = 0.8, *p* = 0.4) and HDI (t=-1.7, *p* = 0.08) but a significant interaction between FLPR and HDI (t = 3.24, *p* = 0.001). Countries with lower HDI displayed a tendency to benefit from increased FLPR, as it reduced male suicide rates. The opposite trend was visible in countries with a higher HDI (Fig. 3).

Results for female suicide rates from model 1 showed no significant main effect of FLPR (t=-0.68, *p* = 0.5) and no significant interaction (t = 1.42, *p* = 0.15), but a significant main effect of HDI (t = 1.98, *p* = 0.049) (Fig. 3).

### Model 2

**Fig. 4:**
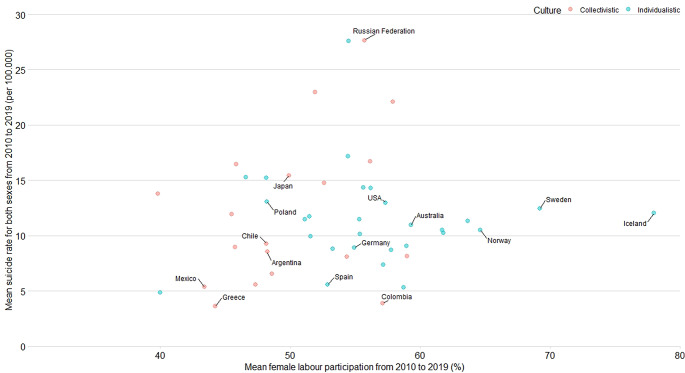
Mean female participation and mean suicide rates for both sexes between 2010 and 2019 by country and individualism/collectivism score

Preliminary visual inspection of the data revealed that individualistic countries (Hofstede’s index score above 50) tend to have higher mean FLPR (Fig. 4).

**Fig. 5:**
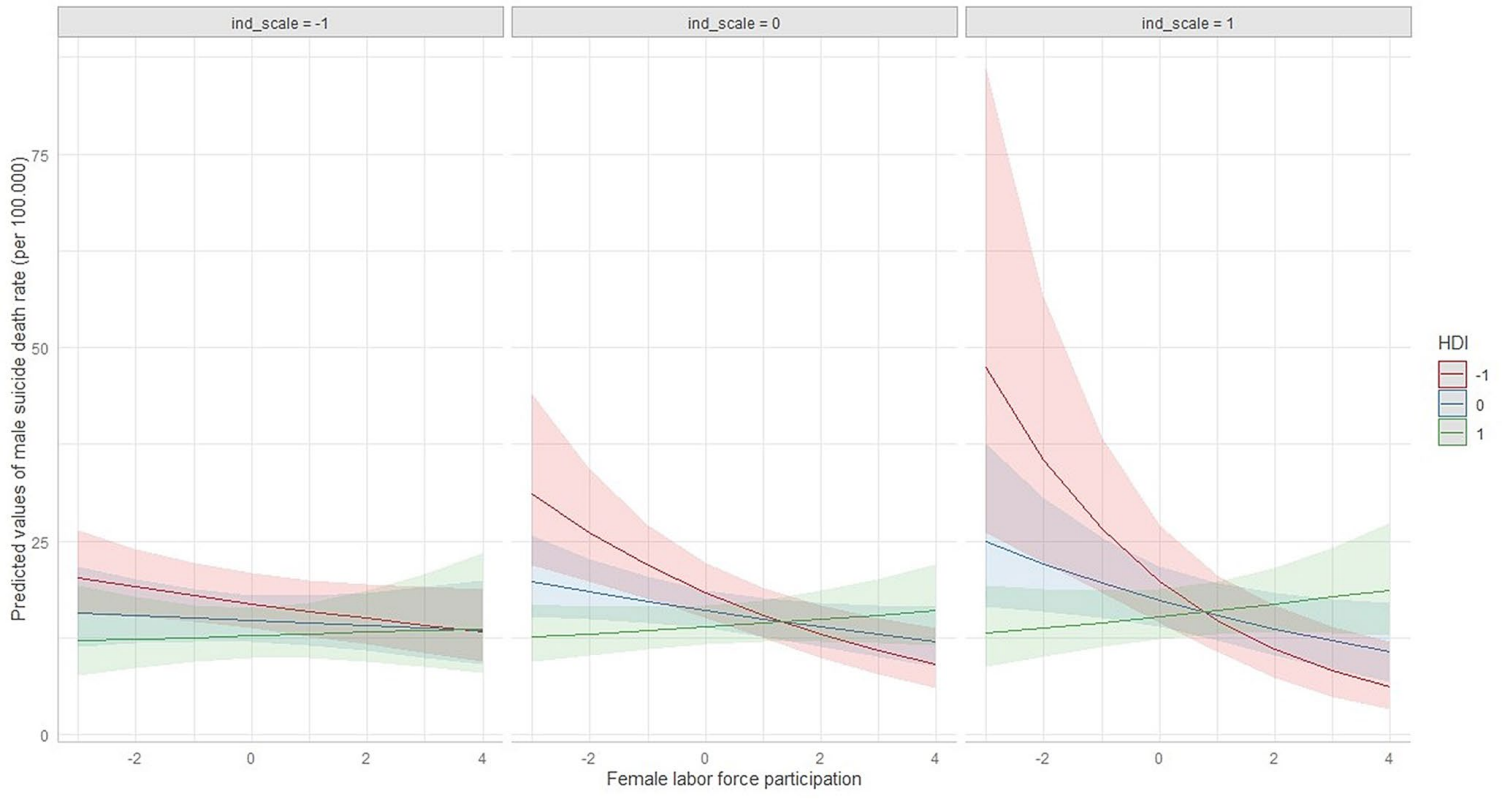
Predicted male suicide rates, by changes in FLPR (scaled), HDI (scaled) and individualism/collectivism score (scaled)

Model 2 resulted, for male suicide rates, in a significant main effect of FLPR and HDI. Moreover, there were significant interactions between FLPR and HDI and between FLPR, HDI, and individualism/collectivism scores (Fig. 5). No significant results were recorded for female suicide rate (Table 3).

**Table 3:** Results model 2

	Estimate	Std. Error	df	t value	*p*-value	
Male suicide rates						
FLPR	-0.07	0.035	286.23	-1.987	0.047	*
HDI	-0.14	0.052	314.88	-2.612	0.009	**
Hofstede’s index score	0.08	0.076	63.51	1.095	0.27	
Time	-0.01	0.004	109.51	-2.501	0.013	*
FLPR: HDI	0.11	0.023	135.46	4.421	< 0.001	***
FLPR: Hofstede’s index score	-0.05	0.033	271.01	-1.457	0.14	
HDI: Hofstede’s index score	0.004	0.038	167.17	0.12	0.9	
FLPR: HDI: Hofstede’s index score	0.07	0.021	193.11	3.09	0.002	**
Female suicide rates						
FLPR	-0.03	0.053	232.40	-0.668	0.51	
HDI	0.07	0.073	194.68	0.941	0.34	
Hofstede’s index score	0.08	0.081	60.842	0.952	0.34	
Time	-0.01	0.005	101.63	-2.552	0.01	*
FLPR: HDI	0.03	0.036	126.99	0.892	0.37	
FLPR: Hofstede’s index score	0.03	0.051	246.67	0.58	0.56	
HDI: Hofstede’s index score	-0.03	0.053	155.41	-0.621	0.53	
FLPR: HDI: Hofstede’s index score	0.01	0.034	178.94	0.458	0.64	

Signif. Codes: *** *p* < 0.001 ; ** *p* < 0.01 ; * *p* < 0.05

### Goodness of fit and intra-class correlation

Goodness of fit showed non-significant improvement between all the models tested for female suicide rates. On the other hand, for male suicide rates, the small but significant increase in AIC values for model 2 indicates worst fit of the most complex model in comparison with the simpler one (Supplementary materials).

Intra-class correlation (ICC) of the models for male and female suicide rates indicated highly clustered data: results were highly dependent on the country to which the observations belong (Supplementary materials).

## Discussion

This study examined the relationships between female labor participation, HDI, Hofsted’s individualism/collectivism index and suicide rates in a diverse sample of countries.

The resulting main aspects are as follows. First, in countries with average and lower Human Development Index (HDI) scores, high female participation in the workforce is linked to a decreasing trend in male suicide mortality. However, in countries with higher HDI, increased female labor participation is associated with higher male suicide rates.

Second, individualism/collectivism scores alone do not significantly impact male suicide trends. However, higher individualism scores seem to strengthen the association between FLPR and male suicide rates (both the negative association in mean HDI and lower HDI countries and the positive association in countries with higher HDI).

Our findings differ from previous studies which showed that increasing participation of women in the workforce may increase suicide rates (Milner et al. 2012; Moore and Heirigs 2021), especially in countries with lower human development (Chen at al., 2017) or showed no to positive effect in countries with higher development (Stack, 1987; Burr et al. 1997; Chen et al. 2017). However, it is important to note that these studies differ from the current paper in their statistical approach. As pointed out by Chen et al. (2012), depending on the type of study, results may differ: while studies using time-series resulted in a positive impact of female labor force participation on suicide rate, studies using cross-sectional methodologies resulted in a negative impact of FLPR.

Contrary to the study of Lenzi et al. (2012) which found increased suicide rates correlating with individualism measured in the general sample, our study showed that individualistic values can have further positive impact on the beneficial effect of increased FLPR on suicide in lower HDI countries.

Contrary to other studies (Fernquist and Cutright 1998; Milner et al. 2012; Chen et al. 2017; Moore and Heirigs 2021), this study did not find an association between female suicide rates and female labor force participation. The positive effect of HDI on women’s suicide rates (Model 1) disappeared once accounting for Hofstede’s index score (Model 2).

### The impact of Human Development Index

In countries with relatively lower and mean human development, female labor participation positively affects male mental health. A possible explanation could be that in these economies, a rise in FLPR, and consequent rise in demand for family-friendly policies, such as parental leave and affordable childcare services, result in greater economic security, reduction of financial stress, and consequent improvement of men’s mental health (Burr et al. 1997; Thevenon, 2011). Equal rights and opportunities boosting female participation can help reduce the burden connected to the expectations of traditional male roles, which might still exist in expanding economies.

Conversely, in countries with higher HDI where we see a rise in male suicide connected to an increase in FLPR, we could advance the hypothesis that as economies keep evolving and social change consolidates, the persistence of outdated gendered values tends to harm men, as well as women, by perpetuating an ideal of masculinity now counterproductive. The disruption of the traditional role of men as breadwinners has been proven to impact relationship satisfaction (Blom and Hewitt 2020) and produce an increase in male psychological distress connected to partner’s income increase (Syrda 2020). In addition, these economies might experience a decline in jobs traditionally dominated by men while those requiring higher education credentials grow. In many highly developed countries, there’s been a noticeable shift in educational achievement, with women outperforming men (Goldin et al. 2006; Esteve et al. 2016). Changes in educational attainment paired with service-oriented economies, which often require higher education credentials, may provide a partial explanation of poor mental health among men.

### The role of individualistic values

Concerning the role of culture, initially, individualistic values prioritizing autonomy and self-determination can foster greater gender equality and increase female workforce participation, along with the associated benefits. However, over time, these values may also exacerbate adverse outcomes for male mental health, as we see in countries with higher HDI. The dissolution of traditional family structures, paired with lower marriage rates and higher divorce rates, leaves men with fewer social integration opportunities outside the family nucleus (Lenzi et al. 2012; Yip at al., 2015). This lack of social support networks can intensify feelings of isolation and vulnerability, particularly in the absence of traditional gender roles (Pampel 1998).

### Strengths and limitations

Study limitations affect the reliability and generalizability of its findings on global suicide rates. First, data availability constrained both the sample size and the types of variables considered. Additionally, potential issues with the dependent variable, suicide rates, might have arisen due to variations in death reporting practices and societal stigma associated with suicide across countries. Nevertheless, these biases are typically stable over time, even when accounting for possible sources of bias in data recording (Jougla, 2002).

Furthermore, the study had an overrepresentation of European and Western-culture countries. The cultural profile of countries with higher HDI (equal or above 0.92) and relatively lower HDI (equal or below 0.81) is somewhat homogeneous. In the first case, countries are mostly individualistic, and, in the latter, they are primarily collectivistic. Therefore, in these groups, inference of the estimated effect of changes in FLPR and HDI, in light of Hofstede’s index score, should be done with caution. It is also crucial to acknowledge the ecological fallacy of epidemiological studies, which involves the potential impact of unmeasured factors (e.g., divorce rate, mental health care access and quality, gender inequality in employment and income) at population level.

The under-representation of Africa and Asia is also a major limitation, as our aim is to provide a global overview of the topic under study. However, selection was based on the quality and completeness of suicide statistics which were a concern in these (Milner et al. 2020). Including data from these countries could potentially enrich the analysis but might also skew the results.

In addition, variations due to country context remain highly significant potentially compromising the generalization of results.

In conclusion, while the relationship between FLPR and male suicide is complex and multifaceted, this study shows that increased FLPR beneficially impacted male suicide rates in some contexts. Moreover, the findings suggest that if a rise in male suicide coincides with increased female labour participation in highly developed countries, targeted suicide prevention measures for men are essential to maintain low male suicide rates while safeguarding the continued upward trajectory of female workforce participation.
